# Zinc in Dermatology: Exploring Its Emerging Role in Enhancing Botulinum Toxin Formulations and Clinical Efficacy

**DOI:** 10.3390/cimb46110717

**Published:** 2024-10-28

**Authors:** Lacey Foster, Jose A. Foppiani, Helen Xun, Daniela Lee, Begum Utz, Angelica Hernandez Alvarez, Maria J. Domingo-Escobar, Iulianna C. Taritsa, Dominika Gavlasova, Theodore C. Lee, Gavin J. Lin, Umar Choudry, Samuel J. Lin

**Affiliations:** 1 Division of Plastic and Reconstructive Surgery, Beth Israel Deaconess Medical Center, Harvard Medical School, Boston, MA 02115, USA; 2 Department of Plastic and Reconstructive Surgery, University of Minnesota, Minneapolis, MN 55455, USA; 3 Izmir Biomedicine and Genome Center, 35340 Izmir, Turkey; 4 Institute of Clinical and Experimental Medicine, 140 21 Prague, Czech Republic; 5 Georgetown University, Washington, DC 20001, USA; 6 Nobles and Greenough School, Dedham, MA 02026, USA

**Keywords:** zinc, botulinum toxin, dermatology, cosmetic science, aesthetic injectables, formulation stability

## Abstract

This literature review provides a novel exploration of zinc’s multifaceted roles in dermatology, with a particular focus on its potential integration into botulinum toxin formulations—an area that remains relatively underexplored in clinical practice. Zinc is widely recognized for its critical functions in skin health, including morphogenesis, regeneration, and protection, and its use in aesthetic medicine offers a unique opportunity for innovation. Specifically, incorporating zinc into botulinum toxin formulations could enhance the efficacy and stability of these treatments. Although zinc has historically been used in topical dermatological products and systemic health interventions, its potential in cosmetic preparations, such as anti-aging therapies or non-invasive aesthetic treatments, remains under-researched. Emerging patents suggest promising formulations combining zinc with botulinum toxin that may improve product stability and extend therapeutic effects. While current studies on oral zinc supplementation present mixed results concerning its ability to prolong botulinum toxin effects, this underscores the need for more rigorous investigation in the realm of aesthetic medicine. Zinc’s well-established role in stabilizing dermatological products, such as sunscreens, and its applications in wound healing and skin regeneration, further highlights its potential for broader therapeutic uses beyond cosmetic applications. This review identifies a critical gap in the literature and calls for future research to optimize zinc concentrations and delivery methods specifically for aesthetic medical procedures, offering new insights into improving dermatological treatments beyond the scope of traditional cosmetic preparations.

## 1. Background on the Properties of Zinc

Zinc is crucial for skin health, participating in skin morphogenesis, regeneration, and maintenance, providing protection and defense. About 6% of the body’s zinc is found in the skin, with higher concentrations in the epidermis compared to the dermis [1]. The recommended daily intake of zinc is 9 to 11 mg for women and men, and 11 to 12 mg for pregnant and lactating women. Zinc supplements come in different forms, each with varying percentages of elemental zinc. Maintaining balanced zinc levels is crucial, as both deficiencies and excesses can lead to health issues [2]. Zinc has been used for centuries in various forms due to its properties. Topically, it is used in preparations like zinc oxide and calamine for sun protection and soothing, as well as in anti-dandruff shampoos. Its applications have grown to include treatments for infections, inflammatory skin conditions, pigmentary disorders, and certain skin cancers. While oral zinc’s role in treating deficiency syndromes is well-known, its significance for infant growth and development has been acknowledged more recently [1]. Zinc stabilizes cell membranes, acts as a cofactor for various metalloenzymes (like MMPs), is involved in superoxide dismutase and metallothionein functions, and participates in basal cell mitosis and differentiation (Vollmer et al.). Mahoney et al. studied the effects of a 0.1% copper-zinc malonate cream on photoaged skin, finding that it significantly enhanced elastin biosynthesis and elastic tissue accumulation in 21 female patients after six weeks of treatment. The study concluded that this bi-metal compound has the potential to regenerate elastic fibers, contributing to wrinkle reduction in photoaged facial skin [3].

**Figure 1:**
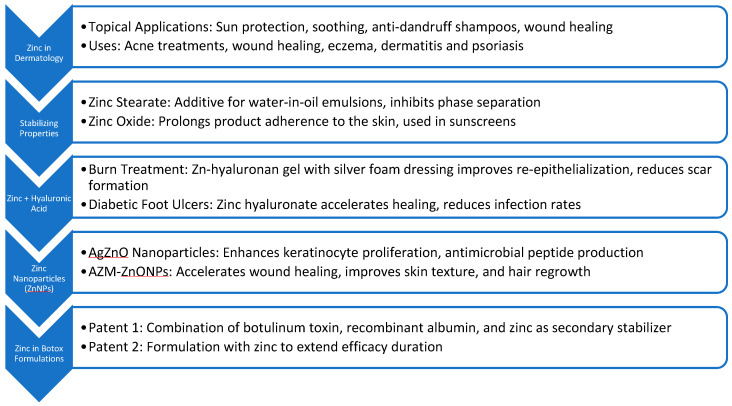
An Overview of the Various Zinc Formulations.

Given zinc’s extensive properties and its crucial role in skin health, this review aims to provide an overview of the uses of zinc in dermatology, focusing on its applications with botulinum toxin and stabilizing properties. By examining current research and clinical practices, this article offers an original perspective on the multifaceted role of zinc in enhancing dermatological treatments and product formulations, shedding light on areas that have been largely overlooked in existing literature (as summarized in Figure 1).

## 2. Zinc in Dermatological Products

Zinc is an essential trace mineral in the human body, and regular consumption is crucial for optimal health. Over the years, zinc has been pivotal in creating treatments for diverse skin conditions due to its wound healing and antimicrobial attributes [4]. It facilitates skin re-epithelialization while curbing inflammation and bacterial proliferation [5]. The release of zinc ions from zinc oxide (ZnO) nanomaterials plays a beneficial role in wound recovery, leading to its widespread use in cosmetics and ointments. ZnO nanoparticles, in combination with chitosan hydrogel, exhibit enhanced antibacterial effects, making them a valuable component in wound dressings [6]. Additionally, zinc oxide (ZnO) has gained traction as a UV shield in physical sunscreens [7]. Notably, microfine ZnO offers superior protection against long-wave UVA radiation compared to its titanium dioxide counterpart and appears less chalky on the skin [8].

In cosmetics, inorganic metals, specifically titanium dioxide (TiO_2_) and zinc oxide (ZnO), are indispensable for crafting opaque inorganic sunscreens. Standard particle sizes of ZnO and TiO_2_ can leave a noticeable whitish cast on the skin, particularly on darker skin tones. This has spurred demand for innovative formulations with finer nanoparticles, typically below 100 nm in size. Such minuscule particles, undetectable by regular microscopes, blend seamlessly on the skin, providing effective UV protection without a discernible residue. Among inorganic UV filters, while ZnO offers broad-spectrum UVA–UVB protection, TiO_2_ is particularly adept at shielding against UVB rays [9].

## 3. Uses of Topical Zinc

One of the most well-known uses of zinc in topical formulations is zinc oxide, which acts as a skin protectant and has astringent, soothing, and protective properties. It is commonly found in diaper rash creams, sunscreens (as a physical UV blocker), and other protective ointments. Zinc has been used in some topical treatments for acne due to its anti-inflammatory and antibacterial properties. Some research suggests that zinc can help reduce the production of sebum, which can contribute to acne. Topical 5% zinc sulphate was found to be effective in mild to moderate acne [10]. Some ointments contain zinc as it may promote wound healing. It is believed that zinc might help with collagen synthesis and immune function, both of which are crucial in the wound healing process. Some creams formulated for eczema and dermatitis might contain zinc oxide and zinc sulfate due to its anti-inflammatory and skin-soothing properties [11]. Some topical treatments for psoriasis may include zinc. Its anti-inflammatory properties can be beneficial in managing psoriasis. For example, topical 0.25% zinc pyrithione applied twice daily was found useful in plaque psoriasis [12]. The applications of zinc in dermatology are extensively discussed in the article by Gupta et al. and briefly shown in Table 1 [1].

## 4. Stabilizing Properties of Zinc

Zinc salt with stearic acid possesses stabilizing property and thus is utilized in many personal care products. This is why zinc stearate is a common additive for water-in-oil emulsions, because it can inhibit phase separation by controlling viscosity. In addition, zinc stearate and zinc myristate are also used as adhesion enhancers in face powders with anticaking, opacifying, and viscosity controlling properties [13].

Zinc oxide, in dermatological applications, prolongs the duration of product adherence to the skin, a trait commonly noted in sunscreens and protective creams. Both titanium dioxide and zinc oxide are the predominant metal oxides employed as UV blockers. Due to their inherent chemical properties, it is essential to coat these two pigments with neutral agents, such as silica, alumina, stearic acid, or silicone mixtures. When exposed to UV light without this coating, the TiO_2_ surface releases excited electrons, leading to the creation of free radicals that can cause oxidative harm to skin layers. Similarly, uncoated ZnO may alter the product’s pH, as it can partially transform into Zn hydroxides, subsequently introducing OH^−^ ions into the mixture. This protective coating also ensures that particles do not cluster together, ensuring a consistent dispersion of pigments. This not only maintains the stability of the formulation but also optimizes its UV shielding capability [14]. Micro or nanoparticulate titanium dioxide and zinc oxide produced by brands such as Z-Cote (BASF Corporation, Antwerp, Belgium) and ZinClear (Advanced Nanotechnology Ltd., Perth, Australia), may provide elegant sunscreen formulations [15].

Aside from cosmetic uses, zinc has been used to stabilize insulin formulations and vaccines. In insulin formulations, zinc ions help keep insulin molecules stable by forming hexameric structures. When left on its own, the insulin monomers are unstable and can quickly cluster into amyloid fibrils. Thus, insulin is primarily formulated as hexamers to prevent insulin aggregation. Novolog (aspart) and Humalog (lispro) are formulated in sodium phosphate buffer with a threefold molar excess of zinc ion, relative to the insulin hexamer. This formulation with zinc ensures stability in the T6 structure, and the hexamer’s breakdown is the determining factor for absorption through the skin and the start of its effects. On the other hand, Apidra (glulisine) does not use zinc but uses the surfactant polysorbate 20 for stabilization [16]. Zinc has also been studied for its stabilizer role in DNA/RNA and protein vaccines, as zinc salts can help maintain the structural integrity of proteins, preventing them from denaturing or aggregating. For example, zinc salts (i.e., zinc oxide, zinc carbonate, and zinc acetate) were found to improve insulin encapsulation efficiency and stability in poly (lactic-co-glycolic acid) (PLGA) microspheres. When insulin was encapsulated with a zinc salt, the protein secondary structure remained unaltered, and no degradation or aggregation products were found [17]. Zinc has been used as an adjuvant in vaccines as it can help create a stronger immune response in people receiving the vaccine [18]. These have primarily been at the research stage and has not led to widespread commercialized vaccines with zinc as an adjuvant.

Zinc is extensively studied for its wound healing and antibacterial capabilities. These capabilities make zinc an ideal candidate especially for chronic wounds, such as burns and diabetic ulcers. Many of these studies incorporate zinc in the form of nanoparticles, often paired with gelling agents like hyaluronic acid or hydrogel materials such as chitosan, heparin, and alginate [17].

## 5. Zinc in Hyaluronic Acid Preparations—Uses in Dermatology

Hyaluronan (HA), also known as hyaluronic acid or hyaluronate, is a polysaccharide of significant molecular mass prominently present in the extracellular matrix, particularly within soft connective tissues. Structurally, HA is a disaccharide polymer, consisting of repetitive sequences of d-glucuronic acid and n-acetylglucosamine [19]. Recognized as a crucial component in tissue regeneration and wound healing, the distinct lengths of HA polymers each play a unique role during different wound healing phases. For instance, larger HA molecules mainly offer structural support, filling spaces, while smaller fragments participate in processes like angiogenesis, inflammation, and immunostimulation [20]. Recently, the properties of HA have gained traction in the field of wound care, with several dressings, such as Hyalofill (Anika), Hyalomatrix (Anika), Ialuset (Laboratoires Genévrier), and Hyiodine (Contipro), harnessing its benefits. Clinical studies suggest that these HA-infused dressings are effective across a range of wound types [20]. Moreover, the strategic use of HA has been linked to improved wound healing and a noticeable reduction in scar formation. This is further evidenced by a double-blind RCT involving breast surgery patients, where a HA sponge combined with zinc (HylaSponge System) resulted in heightened patient satisfaction due to improved scar appearance, although the exact zinc concentration remains unspecified in the study [21].

Chronic wounds, including burns and diabetic ulcers, are intricate skin injuries that often necessitate extended rehabilitation. These wounds are prone to deterioration and infection, underscoring the need for advanced dressing solutions. A recent study formulated a gel integrating carboxymethylcellulose (CMC), hyaluronic acid (HA), and zinc oxide nanoparticles (ZnO NPs). Based on their findings, the optimal manufacturing process employed a 1:4 ratio of HA to CMC gel to achieve the desired viscosity. Various concentrations of ZnO NPs, ranging from 0.05% to 10% by weight, were introduced to the mixture. To pinpoint the ideal composition, the study evaluated physical properties, conducted anti-bacterial tests, and performed a cell migration assay. Samples containing 0.5% to 10% *w*/*v* concentration exhibited potent bactericidal properties against both gram-positive and gram-negative bacteria. Wound healing experiments indicated enhanced cell proliferation with HA/CMC/ZnO gels at weight-to-volume ratios of 0.05% and 1.0% *w*/*v*. In summation, the gel with a 1.0% *w*/*v* ratio emerged as a prominent wound-healing material due to its robust anti-bacterial activity [22].

In a separate study targeting the creation of a preclinical test model for superficial partial-thickness burns, burn injuries were inflicted on 90 male Wistar rats using a soldering tool heated to 130 °C. The investigation compared the efficacy of four treatments: silver-sulfadiazine cream, zinc-hyaluronan gel, silver foam dressing, and a combination of the zinc-hyaluronan gel with the silver foam dressing. Both the zinc-hyaluronan gel and its combination with the silver foam dressing significantly reduced the non-epithelialized area compared to the pharmaceutical-grade silver-sulfadiazine cream. By the tenth day, the combined treatment showed superior re-epithelialization, and by day 22, it resulted in the formation of the thinnest scars. This research, conducted on a rat burn model for therapeutic purposes, concluded that the combined application of zinc-hyaluronan gel with a silver foam dressing was the most effective treatment for wound healing in this context [23].

In a clinical evaluation of the Zn-hyaluronan gel’s potential as a pharmaceutical treatment for partial-thickness burns, a cohort of 60 patients, each with an average burn covering 3% of their Total Body Surface Area, was observed. The exudative phase lasted for three days, with no infections reported. It is important to note that these findings pertain to the therapeutic and pharmaceutical use of the gel for wound healing, not its application in cosmetics or cosmeceuticals. Additionally, animal testing for cosmetic products has been banned under Regulation CE 1223/2009, which strictly prohibits the use of animal data to support the safety or efficacy of cosmetic and cosmeceutical formulations. By the end of the second week, 52 out of 60 patients witnessed wound healing, averaging a complete recovery in 10.5 days. A remarkable 93.3% healing rate was recorded within the observation window. The gel application yielded significant pain reduction by days 5.5 and 6.3 for spontaneous and movement-induced pain, respectively. Notably, the gel formed a protective, flexible layer over wounds, fostering a moist environment crucial for healing. This layer naturally sloughed off as the healing process progressed, solidifying the Zn-hyaluronan gel’s efficacy for partial-thickness burns [24].

Lastly, an open-label RCT delved into the effects of zinc-hyaluronate on neuropathic and neuroischemic diabetic foot ulcers. The study involved 59 diabetic patients, all of whom underwent standard treatments. Among these patients, a subset of 35 with 43 ulcers received both the conventional treatment and zinc hyaluronate, while the remaining 24 with 28 ulcers received only the standard care, acting as controls. Zinc hyaluronate was applied daily in doses ranging from two to four drops directly onto the ulcers. The average healing duration was significantly shorter in the group treated with zinc hyaluronate (74 ± 31 days) compared to the control group (92 ± 25 days). Infections were reported in approximately two-thirds of the ulcers in both groups, necessitating comprehensive antibiotic regimens. The diminished infection rate in the zinc hyaluronate-treated group can likely be attributed to the antimicrobial properties of zinc hyaluronate [25].

## 6. Zinc Nanoparticles (ZnNPs) Without Hyaluronic Acid

Zinc nanoparticles (ZnNPs) possess antimicrobial activity and enhance wound healing. Thus, ZnNPs have been widely studied not only in hyaluronic acid based gels, but also alone or in combination with other agents. For example, a study discovered that silver nanoparticles coated with zinc oxide (Ag@ZnO) boosted the growth and movement of human keratinocytes (HaCaT). As a result, there was a heightened expression of Ki67 and vinculin, a membrane cytoskeleton protein, at the wound’s front edge. Notably, the Ag@ZnO triggered keratinocytes to produce antimicrobial peptides hβD2 and RNase7, enhancing defense against both external and internal Staphylococcus aureus from wounds [26].

Another study assessed the effectiveness of azithromycin-loaded zinc oxide nanoparticles (AZM-ZnONPs) for treating infected wounds. ZnO-NPs were formulated, and azithromycin was integrated into them at different concentrations. Hydroxypropyl methylcellulose (HPMC) acted as the gel base. The healing properties were examined in rats using an excision wound model. The rats’ dorsal skin was prepped, and a 1 cm^2^ wound was created, which was then exposed to a MRSA solution. Following 24 h, the rats were treated twice daily with either AZM, ZnO, or AZM-ZnONPs in a 2.5% HPMC gel. The group treated with AZM-ZnONP showed quicker wound healing, evidenced by a higher wound contraction rate. Additionally, this group had improved skin texture and regular hair regrowth compared to other groups or controls [27,28].

## 7. Background on Botulinum Toxin and Its Formulations

In 2022, Americans invested over $11.8 billion in aesthetic procedures, a figure that has consistently risen [29]. Over 3.9 million cosmetic procedures involving neurotoxins were performed in 2022 [30]. This burgeoning demand is part of a global phenomenon, as ISAPS Global Survey illustrated a clear upward trend in non-invasive treatments, with 14.4 million in 2020, 17.5 million in 2021, and 18.8 million in 2022 [31,32]. These numbers reflect the growing popularity of non-invasive interventions, including chemodenervation, which are increasingly sought after for their minimal downtime and noticeable aesthetic results.

Botulinum toxin injections are neuromodulators used extensively for managing wrinkles, facial asymmetry, dynamic expression lines, hyperhidrosis, and many other conditions [33]. These products include Botox^®^ by Allergan (onabotulinumtoxinA), Dysport^®^ by Ipsen (abobotulinumtoxinA), and Xeomin^®^ by Merz Pharmaceuticals (incobotulinumtoxinA). Their broad acceptance, safety, and efficacy in facial rejuvenation and various muscular modifications have contributed to their widespread adoption. However, the recency of Botulinum toxin used by the public results in a dearth of data on long term sequelae.

While there have been case reports of aesthetic patients developing antibodies (NAbs) against Botulinum toxin, this phenomenon is infrequently reported [34]. Their presence can significantly reduce the toxin’s biological activity, diminishing clinical response [34,35]. This poses a challenge for long-term patient management, and the absence of robust studies makes patient discussion of risks and counselling difficult. Furthermore, the immunogenicity of Botulinum toxin remains under-explored, especially its propensity to stimulate antibody formation in aesthetic applications where treatments are recurrent and may involve varying doses and frequencies [36,37].

Studies of NAbs for non-cosmetic purposes lay the initial groundwork of this review, which have shown that antigenicity of Botulinum toxin preparations is a relevant problem in long-term neurologic treatments [38,39,40]. However, patients receiving Botulinum toxin injected for cosmetics purposes have different profiles, exposure timelines, and anatomic sites; thus, they should be studied directly.

Botulinum toxin, a potent neurotoxin from Clostridium botulinum, has eight serotypes (A–G) that block acetylcholine release, causing muscle paralysis. Its effects typically last three months. It is widely used in medical and cosmetic treatments [41]. There are five FDA-approved BoNTA (Botulinum Neurotoxin-A) drugs. They have the following formulation contents: Botulinum toxin (150 kDa) + nontoxic accessory proteins + excipients (lactose, sucrose, gelatin, dextran or serum albumin and buffer systems) [42]. Current Botulinum Neurotoxin formulations do not include Zinc.

More specific formulations of the drugs are as listed below and shown in Table 2:

- Onabotulinum toxin A (ONA; Botox^®^/Vistabel^®^; Allergan Inc., Dublin, Ireland): The 150 kDa neurotoxin is part of a 900 kDa complex with other proteins (complexing proteins) [42]. Other contents of the formulation include human serum albumin and NaCl.
- Abobotulinum toxin A (ABO; Dysport^®^/Azzalure^®^; Ipsen, Paris, France/Galderma, Lausanne, Switzerland): BoNTA is part of a protein complex, but the size of the ABO complex is unknown [42]. Other contents of the formulation include human serum albumin and lactose.
- Incobotulinum toxin A (INCO; Xeomin^®^/Bocouture^®^, NT 201; Merz Pharmaceuticals GmbH, Frankfurt, Germany): Xeomin stands out in its formulation because its purification process eliminates the complexing proteins from the botulinum toxin complex. Unlike other market-available preparations, Xeomin is purely composed of the 150 kD neurotoxin [42]. Other contents of the formulation include human serum albumin and sucrose. Xeomin has the minimal bacterial protein content among all botulinum toxins available. Additionally, even with repeated high-dose applications, Xeomin does not lead to the creation of neutralizing antibodies. Clinical research indicates that Xeomin is comparable in its effects to Botox. A unit of Xeomin is equivalent to a unit of Botox [41]
- PrabotulinumtoxinA (JEUVEAU Evolus Inc., Newport Beach, CA, USA): JEUVEAU contains accessory proteins in a complex of 900 kDA. One vial contains 100 Units of botulinum toxin type A neurotoxin complex, human serum albumin (0.5 mg), and sodium chloride (0.9 mg) in a sterile, vacuum-dried form without a preservative [43].
- DaxibotulinumtoxinA (Daxxify, Revance Therapeutics, Inc., Nashville, TN, USA): DaxibotulinumtoxinA for Injection (DAXI) is a novel BoNTA product containing highly purified 150-kDa core neurotoxin and is the first to be formulated with a proprietary stabilizing excipient peptide (RTP004) instead of human serum albumin [43]. Other contents of the formulation include histidine, trehalose dihydrate, and polysorbate 20.
- MyoBloc (Elan Corporation, Dublin, Ireland): This is an injectable solution comprising botulinum toxin type B, human serum albumin, sodium succinate, and sodium chloride at about pH 5.6.

## 8. Registered Patents on Zinc in Botulinum Neurotoxin-A Formulations with Zinc

Title: Amino acid and zinc containing botulinum toxin pharmaceutical compositions [44].

Summary: The patent application describes a botulinum toxin pharmaceutical formulation that does not contain animal-derived proteins. This formulation combines botulinum toxin with recombinant albumin and potentially zinc. The composition is designed for treating various diseases and afflictions [44].

Key components and variations of the formulation include:

(1) A primary combination of botulinum toxin (e.g., botulinum toxin type A) and recombinant albumin.
(2) An alternate version that consists of botulinum toxin, NAT (N-acetyl tryptophan), and zinc.
(3) A different embodiment encompasses a botulinum toxin combined with a primary stabilizer and a secondary stabilizer. The primary stabilizer could be a recombinant one, like r-HSA, while the secondary stabilizer might be a metal, such as zinc. Alternative secondary stabilizers can be caprylate (octanoate) or NAT.

Title: Botulinum toxin composition having prolonged efficacy duration [45].

Summary: The invention introduces a botulinum toxin composition that includes both botulinum toxin and zinc. The primary goal is to present a novel formulation of botulinum toxin that ensures enhanced safety and effectiveness and offers an extended duration of efficacy. The addition of zinc to the botulinum toxin composition extended the toxin’s efficacy duration and significantly minimized side effects. This patent emphasizes the innovative use of zinc in botulinum toxin compositions, highlighting its potential to prolong efficacy and reduce side effects.

In a specific embodiment, the effect of varying zinc concentrations on the duration of botulinum toxin efficacy was studied. Botulinum toxin samples with incrementally increasing zinc amounts were prepared and tested on rats. The findings revealed a correlation between the added zinc quantity and the duration of the toxin’s potency. To optimize the extended efficacy of botulinum toxin, the zinc concentration should ideally range from 0.001 mM/100 U to 3.0 mM/100 U per 100 units (U) of botulinum toxin. A more preferred concentration is between 0.001 mM/100 U and 0.33 mM/100 U for every 100 U of botulinum toxin. In terms of the botulinum toxin unit (U), the ideal amount of zinc ranges from 0.01 µM/U to 30 µM/U, with a more preferred range being 0.01 µM/U to 3.3 µM/U [45].

## 9. Literature on Zinc in Botulinum Toxin Formulations and Oral Zinc Supplementation

A comprehensive literature search found no articles reporting the use of zinc injections alongside Botox or Botox formulations containing zinc. However, studies have shown that oral zinc supplementation, when paired with phytase, can enhance toxin effect duration. For instance, Koshy et al. discovered that of their seventy-seven participants, 92% who took a combination of 50 mg zinc and phytase saw an average toxin effect duration increase of approximately 30%. In contrast, there was no notable increase in duration or effect in those who took a lactulose placebo or 10 mg of zinc gluconate [46]. Yet, a commentary on the findings of the Koshy et al. study raised concerns about its design, unclear evidence of patient zinc deficiency, and ambiguity regarding the specific zinc dosages used. The commentary also highlighted potential author conflicts of interest, which could have influenced the study’s outcomes [47]. In a separate study, Shemais et al. evaluated the impact of 50 mg/day zinc gluconate (administered four days before BT application) versus placebo on patient smile types [48]. Their results showed 76.9% prevalence of the commissure smile in the group given 50 mg of zinc gluconate compared to the placebo group (*p* < 0.05). Additionally, there was a 7.7% prevalence of the smile type cusp and 15.4% for the complex type in the zinc gluconate group compared to placebo (*p* < 0.05) [48].

A more recent review of these trials (by Koshy et al. and Shemais et al.) determined that the existing evidence does not advocate for zinc supplementation to bolster the effect of botulinum toxin on facial muscles [49]. Therefore, despite botulinum toxin being recognized as a zinc-dependent metalloprotein, there is no concrete scientific backing for the practice of zinc supplementation to enhance its efficacy on facial muscles. Further randomized clinical trials (RCTs) are required to bridge the knowledge gaps concerning the influence of zinc supplementation on botulinum toxin’s longevity.

## 10. Conclusions

The role of zinc in dermatology is multifaceted, ranging from its critical involvement in skin morphogenesis and wound healing to its increasing use in cosmetic formulations, such as sunscreens and anti-aging treatments. This review has elucidated zinc’s broad applications, particularly in stabilizing products, enhancing UV protection, and accelerating wound healing. Both topical and oral zinc play important roles in dermatological health, offering benefits for conditions like acne, eczema, psoriasis, and chronic wounds. Studies demonstrate the efficacy of zinc oxide nanoparticles and zinc-based creams, particularly in promoting wound re-epithelialization and reducing inflammation, showcasing its pivotal role in skin regeneration.

Zinc’s stabilizing properties, as seen in its use in sunscreens and emulsions, underline its utility in maintaining product integrity and prolonging its effects on the skin. Moreover, emerging research on zinc’s incorporation into botulinum toxin formulations suggests potential for enhancing the longevity and efficacy of these aesthetic treatments. Although zinc has been shown to boost the stability of various formulations, further research is warranted to fully explore its potential in aesthetic applications and to address potential risks associated with its long-term use in cosmetic procedures.

In conclusion, zinc’s extensive contributions to skin health make it an invaluable component in dermatological products and treatments. However, given the complexity of its mechanisms and its varying effects depending on dosage and formulation, continued research is essential to optimize its applications and ensure safety in both medical and cosmetic dermatology.

## Figures and Tables

**Table 1:** Summary of Zinc in Dermatological Products and Uses.

Product Type	Zinc Compound	Uses	Key Benefits
Sunscreens	Zinc oxide (ZnO)	Sun protection	Broad-spectrum UVA–UVB protection, less chalky appearance on skin [8,9]
Topical Creams	Zinc oxide, Zinc sulphate	Acne, eczema, dermatitis, psoriasis, wound healing	Anti-inflammatory, antibacterial, promotes wound healing, reduces sebum production [10,11,12]
Ointments	Zinc oxide	Diaper rash, wound healing	Astringent, soothing, protective properties [11]
Shampoos	Zinc pyrithione	Anti-dandruff	Antimicrobial, anti-inflammatory properties [12]
Antibacterial Wound Dressings or Gels	ZnO nanoparticles	Wound dressings, antibacterial gels	Enhanced antibacterial effects, improved wound recovery [6,7]
Composite Gels (ZnO nanoparticles with a chitosan hydrogel)	ZnO nanoparticles + Chitosan hydrogel	Chronic wound treatment, diabetic ulcers, burns	Enhanced wound healing, antimicrobial activity, maintains moist environment [13]

**Table 2:** Zinc in Botulinum Toxin Formulations and Stabilization [40,41,42,43].

Botulinum Toxin Product	Formulation Content	Stabilization Role of Zinc
Botox^®^ (Allergan)	Botulinum toxin + human serum albumin, NaCl [42]	No zinc inclusion in current formulation
Dysport^®^ (Ipsen)	Botulinum toxin + human serum albumin, lactose [42]	No zinc inclusion in current formulation
Xeomin^®^ (Merz Pharmaceuticals)	Pure 150 kD neurotoxin + human serum albumin, sucrose [41]	No zinc inclusion in current formulation, minimal bacterial protein content
Jeuveau^®^ (Evolus Inc.)	Botulinum toxin type A neurotoxin complex + human serum albumin, sodium chloride [43]	No zinc inclusion in current formulation
Daxxify^®^ (Revance Therapeutics)	Botulinum toxin + RTP004 peptide, histidine, trehalose dihydrate, polysorbate 20 [43]	No zinc inclusion in current formulation
MyoBloc (Elan Corporation)	Botulinum toxin type B + human serum albumin, sodium succinate, sodium chloride [42]	No zinc inclusion in current formulation
Patent 1: Amino acid and zinc containing botulinum toxin pharmaceutical compositions	Botulinum toxin + recombinant albumin + zinc	Zinc used as a secondary stabilizer [44]
Patent 2: Botulinum toxin composition having prolonged efficacy duration	Botulinum toxin + zinc	Zinc inclusion to extend toxin efficacy duration [45]
